# The Marketing Firm and the Consumer Organization: A Comparative Analysis With Special Reference to Charitable Organizations

**DOI:** 10.3389/fpsyg.2020.535793

**Published:** 2020-11-25

**Authors:** Gordon Robert Foxall, Valdimar Sigurdsson, Joseph K. Gallogly

**Affiliations:** ^1^Cardiff Business Schooll, Cardiff University, Cardiff, United Kingdom; ^2^Department of Business Administration, Reykjavík University, Reykjavik, Iceland

**Keywords:** the marketing firm, consumer organizations, charitable organizations, bilateral contingency, managerial work

## Abstract

The accurate delineation of various forms of business organization requires a comparative analysis of their objectives, functions, and organizational structures. In particular, this paper highlights differences in managerial work between business firms and non-profits exemplified by the charitable organization. It adopts as its template the theory of the marketing firm, a depiction of the modern corporation as it responds to the imperatives of customer-oriented management, namely consumer discretion and consumer sophistication. It describes in §2 the essentials of the theory and its basis in consumer behavior analysis, and outlines its unique position as the organization responsible for marketing transactions, based on objective exchange, competitive markets and prices, and the deployment of the entire marketing mix. §3 deals in greater depth with the objective, strategic functions, and organization of the marketing firm in terms of the concepts of metacontingency and bilateral contingency. §4 discusses how the marketing firm differs from charities in terms of goal separation, market-based pricing and competition, the entrepreneurial (strategic) process, the pursuit of customer-oriented management, and organizational structure. Particular attention is accorded the organizational differences between marketing firms and charities, which arise as a direct consequence of the distinct patterns of contingency they entail. §5 discusses the implications of the foregoing analysis and draws appropriate conclusions.

## Introduction

Customer behavior is nowadays marked by a high degree of choice and sophistication, and a highly competitive market-place, all of which compel firms to adopt a strategy that has become known as customer-oriented management (e.g., Huber et al., 2001; Lambin and Schuiling, 2012; Kotler and Keller, 2015). There are important differences in managerial work between marketing firms, whose mission is to respond to the “imperatives of customer-oriented management” in order to fulfill their own corporate objectives, and consumer organizations, which pursue alternative objectives. These differences have implications for (a) the relationships that internally link members of the firm or consumer organization, and (b) those that link the firm or consumer organization to its external publics. The nature of these relationships reflects the organizational mission involved and the approach to strategic management and entrepreneurial policy enjoined upon the business by its publics. Moreover, these intra- and extra-organizational relationships can be advantageously analyzed through the concept of bilateral contingency, which is a central component of theory of the marketing firm (Foxall, 1999, 2018, 2020a; Menon, 2020). Although this theory pertains to a specific kind of business organization, that which responds to the imperatives of customer-oriented management, it cannot be defined and analyzed in isolation from other businesses. The consumer organizations of interest include, in addition to charitable organizations, social marketing campaigns, purchasing and marketing co-operatives, partnerships, and public corporations. The relevance of the theory to the analysis of managerial work in marketing firms and consumer organizations depends on an appreciation of its nature as an economic-psychological construal which draws upon behavior analysis, microeconomics, and marketing science to portray the modern corporation. The nature of the marketing firm can be specified only through this inter-disciplinary synthesis. The overriding goal of the present paper is to achieve a clearer understanding of the place and role of marketing in the theory of the firm by considering the similarities and contrasts between the marketing firm and other forms of enterprise. The paper also has several subordinate goals.

First among these is to complement the analysis of consumer behavior that has taken place through the disciplinary lens of consumer behavior by presenting a theory of the firm in similar terms and evaluating it. The aim therefore is also to ascertain the extent to which the amalgam of behavior analysis, behavioral economics, and marketing science that forms the basis of consumer behavior can present a theory of the firm which complements what we know of consumer behavior from this source. Second among the subsidiary goals is to demonstrate the necessity and form of a comparative analysis of the marketing firm compared with other businesses. The main goal requires a broader perspective than that of the firm itself. It must arrive at its conclusions through an understanding not only of the nature of the firm but also of other businesses, consumer organizations such as non-profits, co-operatives, partnerships, and state-owned enterprises. While the different types of consumer organizations would benefit from review, this paper concentrates, specifically, on the business firm in contrast to the charity organization (see Foxall, in preparation for a broader analysis). How these differ in terms of goals pursued, the role of the market (prices determination, competition), the nature of managerial work with respect to entrepreneurship and strategy, organizational structure, and the implications of marketing-oriented management. Third is to exemplify the nature of managerial work in the marketing firm, again through comparative analysis of strategic goals and behavior of the firm and the charity. The emphasis here is on the strategic behavior of the marketing firm, its entrepreneurial mission. This is found to be lacking in consumer organizations. Finally, the paper seeks to develop the theory of the marketing firm by considering the nature of contemporary businesses compared with consumer organizations such as non-profits, co-operatives, partnerships, and state-owned enterprises in terms of their objectives, functions, and organizational structures.

These themes are topical and relevant in light of the sheer number of theories of the firm that are becoming available, many of which ignore marketing and, especially, the imperatives of customer-oriented management. Yet these considerations *define* the modern business firm in the context of the imperatives of customer-oriented management that have been alluded to. We need to understand this better through comparative analysis – hence inclusion of the charity. Another note of relevance is struck by the fact that many of the consumer organizations mentioned are increasingly referred to as “firms” with no distinction made between their objectives, functions, organization and that of the paradigmatic business firm. This is a source of confusion that cries out for understanding and clear methods of demarcation.

## The Marketing Firm: Theoretical Background

### Consumer Behavior Analysis

**FIGURE 1:**
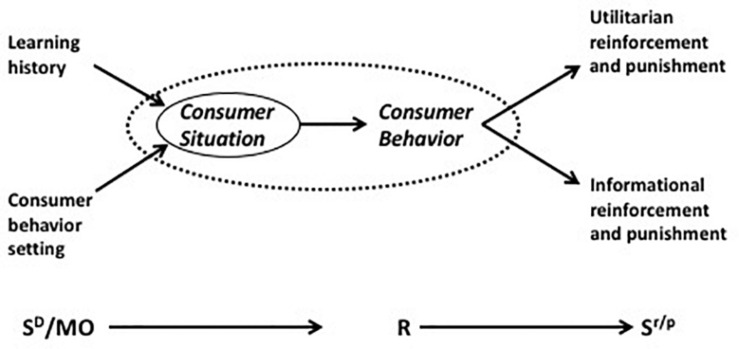
Summative behavioral perspective model; Foxall (2020a).

The theory of the marketing firm proposes a view of the firm as a response to the imperatives of customer-oriented management which is based on an inter-disciplinary framework of conceptualization and analysis. The components of this explanatory system are the approach to behavioral psychology known as behavior analysis, the school of behavioral economics founded on a fusion of behavior analysis and microeconomics (Hursh, 1980, 1984), and the empirically based marketing science of Ehrenberg (1988) and his colleagues. Consumer behavior analysis (Foxall, 2001, 2002). has been described, both theoretically in terms of the empirical evidence that it has inspired, in Foxall (2017) and, accordingly, will be only briefly portrayed here (for more examples of research using consumer behavior analysis, see Sigurdsson et al., 2009, and Foxall et al., 2006). In behavior analysis, a response, R, is explained by reference to pre-behavioral stimuli that set the occasion for its performance (discriminative stimuli or S^*D*^), and the rewarding or *reinforcing* stimuli that have followed this response on previous occasions (S^*r*^). This paradigm, the so-called “three-term contingency,” comprises the explanatory device employed by operant psychology (e.g., Skinner, 1974). An operant response is simply one that *operates* on the environment in order to effect consequences that influence the rate of its subsequent performance. Not all of these consequences are rewarding or reinforcing; some, which lead to a diminution in the rate of responding, inhibit or punish the behaviors they follow and are known as *punishers*. We can, therefore, state the three-term contingency as S^*D*^: R S^*r/p*^ where SD is a discriminative stimulus, R a response, and Sr/p a reinforcer or punisher. The colon suggests a probabilistic relationship between the setting variable and the response, while the arrow denotes a determinate outcome. The consumer behavior setting consists, in addition to SDS, of motivating operations (MO) which are pre-behavioral stimuli that enhance the relationship between a response and its reinforcing consequences (e.g., Fagerstrøm et al., 2010). The depiction of consumer behavior in these terms has led to the formulation of the Behavioral Perspective Model (BPM) of consumer choice (Foxall, 1990/2004, 2016a,b,c). In it, the *consumer situation*, comprising setting variables weighted by the consumer’s learning history, is the immediate precursor of consumer behavior (Figure 1). This interaction of situation and behavior rests upon the consumer’s prior experience (consumption history) which primes the setting stimuli, as a result of which particular behaviors become more probable while others are inhibited (see e.g., Sigurdsson et al., 2015).

Hursch’s approach to behavioral economics relies on the similar functions played by the variables included in behavior analysis and those of neoclassical microeconomics. A response is an act of purchase, consumption, or work while a reinforcer is a positive outcome of these behaviors (a product or service or a wage), and the relation between them is expressed by a schedule of reinforcement in behavioral psychology and a price in the realm of economic behavior. This approach has been employed in research designed to test the BPM. Importantly, recent investigations have established what it is that consumers buy and consume. On the surface of course it appears evident that they purchase and use the products and services they acquire in exchange transactions. The BPM proposes that they consume reinforcers that supply both functional and social benefits, known, respectively, as utilitarian and informational reinforcements. One strand of the behavioral-economic research derived from the model has employed the Cobb-Douglas utility function, which proposes that consumers purchase combinations of products or attributes which maximize their returns or utility within the confines of their income or budget constraint (Douglas, 1976). A series of empirical investigations indicates that what consumers actually maximize is combinations of utilitarian and informational reinforcement (Oliveira-Castro et al., 2015, 2016; Oliveira-Castro and Foxall, 2017). The kind of aggregate data on consumers’ patterns of consumption revealed by the work of Ehrenberg (1988), based on sophisticated consumer panel evidence, has made available the means of testing both the model and the economic hypotheses drawn from it among large representative samples of buyers.

### Delineating the Marketing Firm: The Marketing Transaction

The marketing firm is defined in terms of the kinds of transaction into which it enters with customers and suppliers, *marketing transactions*, which have four characteristics. The first is literal or objective exchanges of products or services, usually for pecuniary benefit; the second is that the exchange occurs in competitive markets and at market-generated prices; therefore third, that these are pecuniary markets: barter could conceivably mark a marketing transaction but this is so rare in normal corporate-consumer dealings as to make pecuniary markets a defining factor; and, finally, the transaction involves the entire marketing mix.

#### Literal or Objective Exchange

A transaction is “the creation of value by voluntary co-operation between two or more economic actors” (Spulber, 2009b, p. 12). The value so created is the benefits to the parties minus the costs of transacting. It is not only firms that incur transaction costs; consumers do so both when they exchange goods directly and when they create and run organizations like clubs, cooperatives, nonprofits, and basic partnerships. These administrative governance costs arise in the course of communicating, processing information, searching, matching, bargaining, moral hazard, adverse selection, free riding, and contracting (Spulber, 2009b, p. 13). Part of the raison d’être of the firm, including the marketing firm, is to make possible transactions for consumers whereby they encounter lower transaction costs than would otherwise be the case. In order to succeed, therefore, a firm has to enable transactions at lower costs than those consumers incur through direct exchange. Firms whose managers believe they can accomplish this, therefore, have an incentive to create markets and an organization. A market, i.e., a means of bringing buyers and sellers together, is created by a firm by designing institutions of exchange to ensure more efficient transactions (Spulber, 2009b, pp. 12—13). But to this we would add that while Spulber sees these functions as those of the firm, the actual creation of markets is a cooperative venture between firms and consumers who are willing to enact transactions with one another. Firms may generally take the initiative but consumers are active participants to any transaction and sometimes the initiative itself also lies with them. By conducting transactions in this way, the firm fulfills what Drucker defines as its very purpose, the creation of a customer: in fact we would further add, the creation of a *consumerate*, a body of consumers who return to purchase the firm’s output sufficiently frequently to allow it to achieve its sales and profit objectives.

**Box 1. Meaning of “consumerate”**

This paper employs the terms customer-, consumer- and marketing-orientation interchangeably throughout the paper. The “consumerate” encompasses the customer base of the marketing firm, be it composed of an aggregation of individual final consumers or a number of corporate customers. Its members are referred to as “customers” *or* “consumers,” these terms being treated as equivalent (Foxall, in preparation).

Marketing transactions entail literal exchange or transfer of legal title. A marketing transaction comprises mutual reinforcement based on literal exchange. In a marketing relationship based on economic exchange, the mutual reinforcement is typically accomplished by an item-for-item switch of valued items. The requirement that marketing transactions be understood as literal transfers entails that marketing firms operate in pecuniary markets. Each party to a marketing relationship provides the other with utilitarian and informational reinforcement: typically, goods which supply functional and social utilities are traded for money and marketing intelligence. The marketing intelligence provided by customers, information about what they have bought and their experience of it and their plans for the future provides informational reinforcement which guides the marketer’s strategic planning and marketing management activities. The literalness of exchange in typical pecuniary trading is easily discerned but the question arises what is actually exchanged in the case of intangibles such as services: the essence of a marketing transaction is mutual transfers of legal title to a product or the outcome of a service. Such exchange is a transfer of property rights (Commons, 1924; Demsetz, 1995; Posner, 1995). Ownership, in the sense of legal entitlement, and contractual requirements are elements of the contingencies of rewards and penalties which can alter behavior, just as the market itself is ultimately a source of mutually acknowledged and reciprocally binding contingencies (Foxall, 1999).

**Box 2. Meaning of “marketing transaction”**

A *marketing transaction* is marked by (i) objective exchange which occurs in a (ii) competitive market, according to (iii) market-generated prices, and (iv) employing the full marketing mix of product, price, promotion and place. This is a contractual relationship between two or more free-acting parties (Foxall, in preparation).

Marketing transactions are invariably accompanied by other, less tightly defined relationships, known in the theory of the marketing firm as *mutuality relationships* (Foxall, 1999). These are social relationships which are characterized by reciprocally contingent reinforcement but which do not involve literal exchanges. They are not economic in nature and do not involve marketing exchanges. They might include, for instance, informal communications between a salesperson and a customer, possibly in the form of a social gathering organized by one or other party, which facilitates mutual interaction (“getting to know you”). They would also include more formal relationships in which a salesperson canvassed a prospective customer or a potential buyer requested product information from a marketing firm.

**Box 3. Meaning of “mutuality relationship”**

A *mutuality relationship* is a (i) socially based, (ii) non-contractual, and (iii) non pecuniary relationship; it may involve (iv) informal competition for resources, time, etc., but (v) carries no marketing considerations. Mutuality relationships do not involve objective exchange but they do entail reciprocal reinforcement. Such relationships may characterize interactions among members of a single organization or those that facilitate more formal exchanges between organizations (Foxall, in preparation).

#### Competitive (Pecuniary) Markets and Prices

For the most part, public bodies are monopolies or work amicably and in accordance with similar organizations. In contrast, firms are rivals with strategic goals and are under the constraints of antitrust legislation, unless authorized as a joint venture. These firms require the open market if they want to pursue marketing orientated management. This allows the firm to decide, using analyses of current consumer behavior and projections of future consumer behavior, what its sphere of operations will be. According to Spulber (2009b), firms require this freedom, though some organizations, which often claim to be marketing practitioners are unable to engage in marketing-oriented management in the ways discussed in this paper. Social marketing campaigns, for instance, are not firms in Spulber’s sense because the objectives of the organizations involved do not differ from those of the owners/members. Bodies engaged in “social marketing” do not have a product or service which is literally exchanged in pecuniary markets, *or* do not use a competitive price mechanism. By and large, they have an amorphous output such as “smoking reduction” (rather than a concrete product or service that can participate in legal transfers based on financial consideration). Public organizations are often influenced primarily by state interventions rather than being able to set prices or determine the focus of their business in an autonomous fashion. Furthermore, public enterprises do not necessarily compete for consumers, nor necessarily set prices; nor yet determine for themselves the business they are in on the basis of market considerations. Their scope for effecting free exchanges in unrestricted markets is highly limited to the extent that they are directed by interventionist government policies.

#### Deployment of the Whole Marketing Mix

The whole marketing mix, not any specific part of it, generates sales. The marketing mix is a model, centered on product, place, promotion and price, used to pursue objectives related to influencing demand for brands (Borden, 1964). Although there are examples of firms enhancing their advertising budgets and thereby increasing their sales, perhaps dramatically, it is not the case that “advertising creates sales,” as though it existed in a vacuum that excluded product, price, and distribution utilities. The essence of marketing as a subject area that is distinct from economics, sociology, and psychology inheres in its adoption of the brand as its distinctive level of analysis. This goes beyond the product which suffices for other disciplines. The brand, over and above the product, is a matter for the entire marketing mix to create, communicate, and sustain. No element of the mix – neither product, place, promotion, or price – can be omitted in the quest to create a customer. The marketing firm, therefore, by definition employs the entire marketing mix.

## Objective, Functions, and Organization of the Marketing Firm

### Objective

Spulber (2009a, b) argues that the firm differs from other commercial and social organizations (also described as *consumer organizations*) in having a goal that is different from that of its owners, namely profit maximization. Firms’ owners, he says (though other stakeholders may fall into the same category: Fama, 1980; Mäntysaari, 2012; Foxall, 2020a), have a separate goal: while they may *desire* to associate with a profit maximizing organization, their own goal is consumption. Spulber (2009b, p. 63) separation criterion is stated repeatedly in his text, though never more forcefully than in the judgment that a firm is “a transaction institution whose objectives differ from those of its owners.”

It can be argued, moreover, that while profit is undoubtedly important to the marketing firm not immediately of interest to its stakeholders who remain essentially consumers, it is not *maximal* profit that is the marketing firm’s concern. Apart from the well-known difficulties of measuring the phenomenon of maximized profit and of knowing when it had been attained, the firm would require to know its marginal costs and revenues at each level of production, both those achieved and those potential, a requirement that for most large-scale organizations is not feasible (e.g., Kahneman et al., 1986). Rather, it seems likely that the marketing firm can be best regarded as seeking to maximize sales revenues subject to a minimum profit constraint, as Baumol (1967) proposed. Sales revenues are more tractable when it comes to the firm’s knowing where it stands, means of increasing them are available in the various elements of the marketing mix, and as long as sufficient profit is earned to satisfy shareholders and reinvest in the business, the firm’s tasks of information gathering and processing are much more straightforward than is the case for the theoretically elegant but impracticable goal of profit maximization (Hall, 1967).

Sales revenue maximization seems a more realistic goal to ascribe to the marketing firm as we have defined it for several reasons. First, sales maximization nicely combines maximization with satisficing (in the form of the minimum level of profit required for the survival of the firm). This is not the amorphous “psychological and emotional” satisficing that Demsetz (1995, p.78) speaks of. Rather, it is as precise as human wit can make it given that information is not perfect and cognitive judgments are boundedly rational. The profit level aimed at is that which satisfies shareholders and leaves room for reinvestment in the business. This is as measurable as it gets. Second, it retains Spulber’s intent in that it is not an objective shared by the owners or other stakeholders. Stakeholders may *prefer* a sales maximizing firm that can adequately compensate shareholders and reinvest but their objective remains consumption. Third, it is an aim which the directors can more nearly approximate than is profit-maximization since it is easier to detect how to increase sales at any point than profits since this requires knowing costs as well as revenues. This modified separation criterion – the “sales—profit objective” – captures Spulber’s determination to distinguish the firm from various categories of consumer but is more in accord with the parameters of corporate performance that directors and managers are capable of conceptualizing and calculating.

### Functions

#### The Management of Strategic Scope

**FIGURE 2:**
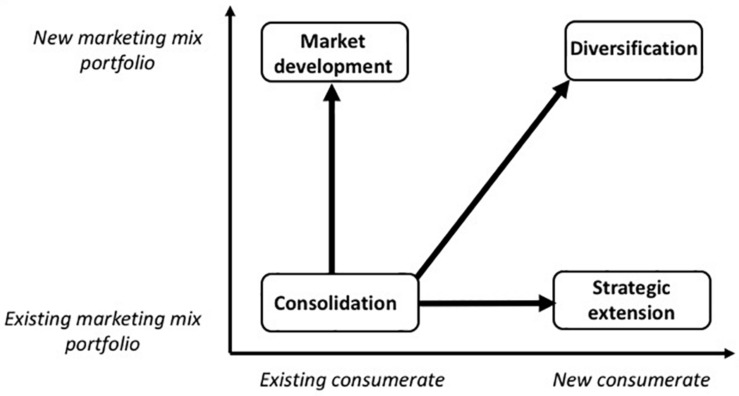
Strategic Scope defines the marketing mixes the firm can support and the consumerates it can serve with them; Foxall (2020a).

The managerial work of the marketing firm consists in (i) the creation of marketing intelligence, (ii) designing a marketing strategy, and (iii) the implementation of this strategy through the management of a portfolio of marketing mixes (Foxall, 2018, 2020a). As a whole, these activities comprise the management of the strategic process and determine the firm’s *strategic scope*, i.e., the range of marketing mixes it can support and the spectrum of consumerates through whose satisfaction it can achieve its objectives of survival and sales maximization (Figure 2).

Three demands, each requiring informational inputs and decision outputs, must be fulfilled by marketing firms: the *Creation of Marketing Intelligence* which typically involves market search and managerial response, reveals the potential (*feasible*) strategic scope of the firm given capabilities and resources; the *Formulation of Marketing Strategy*, concerned to determine the planned strategic scope of the firm, namely the markets it will serve, the marketing mixes with which it will seek to accomplish this, and expectations of further diversification and innovation, i.e., the determination of the *potential* strategic-scope of the firm, and *Marketing Mix Management*, the creation and implementation of the portfolio of marketing mixes through which the firm will seek to achieve its objectives developed in the course of the first two stages. This last determines the *revealed* or *effective* strategic scope of the firm.

Before elaborating the procedures involved in these resource-based operations which constitute the firm’s *strategic process*, we reiterate the concept of *marketing incompleteness* (Foxall, 2020a). Bylund’s (2016) theory of the firm argues that incompleteness involves splitting a productive stage generating partly finished goods for which there is no market: the producer has either to obtain partly completed inputs in order to complete it in-house or find a market for a partly finished product which does not readily exist. This is the source of what he calls the specialization deadlock since extra-market specialization results in uncertainty, a state of affairs which accords with Adam Smith’s statement that the division of labor is limited by the extent of the market. Bylund is predominantly interested in the incompleteness of *productive* processes.

*Marketing* incompleteness is of a different kind. The strategic scope of the firm depends on the assets, productive *and* marketing, at its disposal. Such assets have value only if they can be protected from plagiarism by competitors. The tasks they entail may be undertaken as discrete operations but they are continuous rather than disjointed, and their holistic management is vital to the firm’s strategic planning and determination of strategic scope. They therefore must be confined (kept within an organizational boundary), classified (available only to trusted and interested parties), and their implementation in the market strictly controlled. Marketing incompleteness is revealed and exploited by means of the deployment of these assets and the creative uses the firm makes of them. Market incompleteness is a gap in the market revealed through market search, evaluated by the application of marketing intelligence within the strategic scope of the firm, and responded to through the application/extension of the strategic scope leading to effective marketing mix management which makes clear the actual strategic scope of the firm.

**FIGURE 3:**
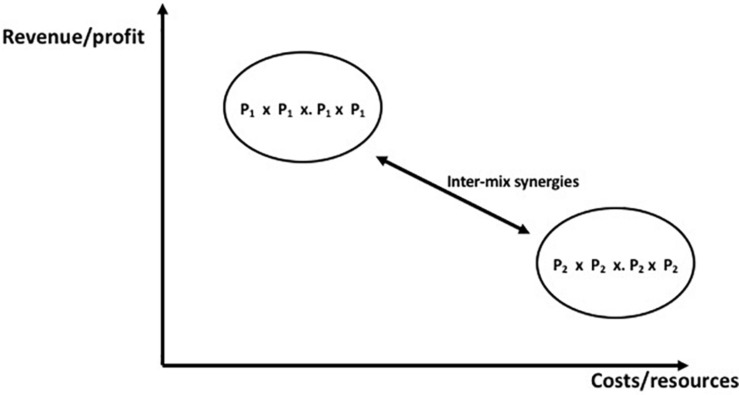
Portfolio of marketing mixes. The strategic scope represented by the portfolio of marketing mixes which the firm manages determines the extent to which the firm consolidates its market positions or diversifies into novel areas. The portfolio must be managed as a single entity. For simplicity, only two marketing mixes are shown; Foxall (2020a).

Possible incompleteness in the market is revealed by market search (confined necessarily within the strategic scope of the firm, though one would hope with an eye to extraneous opportunities too). By revealing the feasible strategic scope of the firm, this stimulates planning based on the fact that market incompleteness is revealed as a gap in the market which can be filled by product development, market development, or diversification. There may be no response necessary to the intelligence so gathered and evaluated. The creation of marketing intelligence presents the *feasible market scope* of the firm. Having established in this way the opportunities open to it, the firm can assess its strategic environment by considering the marketing opportunities available to it and the behaviors of consumers and competitors. This could be done with the aid of external consultants and agents. However, planned movement in any one of these directions reflects a change in the in the strategic scope of the firm (following on from requisite decision-making on the basis of marketing intelligence and planning). This decision-making must recognize that, although market search was undertaken within the strategic scope of the firm its results and revealed potentials have now got to be rigorously reexamined within the capabilities framework of the organization (Day, 2011). But the planning of future marketing scope must not be confined within the preexisting corporate policy and strategy: it ought also to impinge on and challenge that strategic position so that it is not a static straightjacket; if necessary the firm’s strategic scope must be modified and decisions made with respect to the resources the firm will employ and how it will utilize them. The firm which has formulated its strategy is in possession of a clear understanding of its *planned strategic scope* (Anderson, 1982). It is now ready for implementation. The firm must design each of its marketing mixes as a unity of product, place, promotion, and price. It is the marketing mix that produces sales, not just any one part of it. It is feasible that external consultants or agents could assist in this stage. The firm must also manage its portfolio of marketing mixes as a single entity (Figure 3). This eventuates in the *realized strategic scope* of the firm. Hence, Marketing Mix Management is a more complex task than “product portfolio management” (see Cooper et al., 1999) which entails but one element of the marketing mix (Foxall, 2015a). The marketing firm is the vehicle for identifying and responding to market incompleteness, in the course of which its strategic scope is inevitably enhanced through product or market development, and diversification. Marketing firms undertaking this by, first, economizing on transaction costs and, second, increasing sales (physical) and revenues (cash).

Entrepreneurship is involved in all three of these marketing operations, viewed in terms of both the tasks that they entail and the (marketing) resources they command. Entrepreneurship may be defined as, first, the identification of market incompleteness, second the response to it in light of the firm’s current strategic scope, and, third, the deployment of appropriate marketing mixes which ensure that the firm’s overall mix portfolio achieves its sales and profit objectives. This does not necessarily mean it maximizes profit, only that it achieves sufficient profit to enable it to invest, satisfy shareholders, and survive and prosper. Entrepreneurship, then, is the successful planning of a sufficiently profitable feasible strategic scope, and the implementation of the decisions that ensue from this (for research on the Marketing Firm in entrepreneurship see, e.g., Fagerstrøm et al., 2020; Haddara et al., 2020). This process – the strategic or entrepreneurial process – requires. for its inauguration and implementation, an organization which encapsulates the requisite competences, namely the marketing firm. In summary, the management of strategic scope views the strategic process as a single entity, rather than three disjointed spheres of operation, which as a whole is concerned with the creation and implementation of the strategic scope of the firm. Its goal and content is the portfolio of marketing mixes which constitute the emergent output of the business organization which influences, first, consumer behavior and, second, the fortunes of the firm itself and hence its subsequent behavior. Indeed, the management of a whole portfolio of marketing mixes in a unified and harmonious manner is the very embodiment of the firm as a metacontingency and it rests on the concept of the bilateral contingencies that define marketing and mutuality relationships within and beyond the firm.

#### Marketing Assets and Entrepreneurial Encapsulation

In the performance of these functions, the marketing firm internalizes the specialized *marketing* assets it requires to cosmpete successfully: these may be largely intellectual but they are specialized assets nonetheless, and perform the same function in a theory of the firm as the physical assets of production. After all, von Mises comments, in the case of production, that it “is not something physical, material, and external; it is a spiritual and intellectual phenomenon. Its essential requisites are not human labor and external natural forces and things, but the decision of the mind to use these factors as means for the attainment of ends. What produces the product are not toil and trouble themselves, but the fact that the toiling is guided by reason” (von Mises, 1949/2016, pp. 141–2). If this is true of production, it is all the more so of marketing – at least as far as the designation intellectual is concerned, though “spiritual” might be a value-judgment too far in both cases! The strategic scope of the firm depends on the assets, productive and marketing, at its disposal, have value to the firm only if they are protected from competitors. The tasks they entail may be undertaken as discrete operations but they are continuous rather than disjointed, and their holistic management is vital to the firm’s strategic planning and determination of strategic scope. They therefore must be confined (kept within an organizational boundary), classified (available only to trusted and interested parties), and their implementation in the market strictly controlled. Marketing incompleteness is revealed and responded to by means of the deployment of these assets and the firm’s response to them. Marketing incompleteness is first identified via market search, evaluated by the application of marketing intelligence within the strategic scope of the firm, and second exploited by application/extension of strategic scope leading to effective marketing mix management which reveals the actual strategic scope of the firm.

The specialized marketing *assets* integral to this process consist in the information, intelligence, and knowledge. Hence, the specialized marketing *resources* integral to the strategic process are human and, fundamentally, intellectual: the entrepreneurs/managers responsible for the discovery of marketing opportunities and for their planning and implementation. Each of the stages that comprise the strategic process has its own intellectual requirements, the outcomes of which feed into the succeeding stage or the reiteration of the process: *Market Information*, which is data with respect to e.g., behavior of consumers; *Marketing Intelligence* which is market data contextualized within the framework provided by the firm’s strategic capacities and present strategic profile; and *Strategic Knowledge* which is marketing intelligence which leads to the formulation of the potential strategic scope of the firm.

Bylund’s (2016) argument is that the specialization of (production) tasks is only achievable within a firm to ensure that there is a “market” or productive use for the output of subtasks, the theory of the marketing firm emphasizes the organizational implications of the specialization of tasks reliant on strategic marketing information. These might also be readily subdivided on the basis of a novel division of labor but this is not the dominant import for the marketing firm. Such knowledge and the intelligence on which it depends needs to be subdivided and re-combined and managed holistically within a particular strategic vision and this state of affairs is likely to be attainable only within the limits of a particular organization. Management of the actual and tacit knowledge involved is achieved only within an organized framework of managerial control in which top management assumes responsibility for the development of a marketing-oriented management *culture*. The sole element in the process that can advisedly involve extra-corporate inputs is the (relatively routine) gathering of market data. The further the firm advances through the strategic process, the more specialized the assets it produces become, and the greater the need of their corporate encapsulation. Thereby is provided the rationale of the contemporary firm, the marketing firm.

### Organization

#### The Marketing Firm as a Metacontingency

It is rare in the marketing literature for consumer behavior on the one hand and corporate marketing behavior on the other to be specified and explained in similar terms. An aim of the theory of the marketing firm has therefore been to propose a model of corporate response to consumer behavior within the inter-disciplinary framework on which consumer behavior analysis is founded. This has an important consequence for the way in which corporate behavior is portrayed since behavior analysis is traditionally concerned only with the behavior of individual organisms, both human and nonhuman. Any such organism, the behavior of which can be predicted by reference to the consequences that have previously followed responses (its learning history) together with the environmental stimuli that prefigure the kinds of consequences that will ensue from its imminent behavior (its behavior setting) can be termed an *operant system* or a *contextual system* (Foxall, 1999, 2016c). This designation clearly applies to the consumer. However, as the theory of the marketing firm has always proposed (Foxall, 1999), it may also be understood as a contextual system since its corporate behaviors are controlled by the reinforcing and punishing outcomes they incur. While individual’s behavior may be understood through the lens of the three-term contingency – ideally within an experimental setting – explaining that of an organization in these terms requires a leap in conceptualization that comprehends its structure as a system of what Biglan and Glenn (2013) refer to as *interlocking behavioral contingencies* (IBCs).

The supra-individual behavior of the organization viewed as a unit may be inferred from its generating consequences or outputs *over and above the aggregate behaviors of its members* and the effects of the organization’s behavior, *which is greater than the sum of its parts*, on *its* subsequent conduct. The relationships between IBCs, their products (or outputs), and the rewarding or punishing consequences enjoined by their external environments on these products, are known as *metacontingencies* (Biglan and Glenn, 2013). Hence, the supra-personal behavior of the marketing firm consists in the marketing mixes that it generates, launches into the marketplace, and subsequently manages through their life cycles. The accent which the theory of the marketing firm places on exchange relationships as central to marketing transactions suggests the mechanism by which the marketing firm and its customer base are bound together via bilateral contingencies. The metacontingency concept is a means of describing the interactions of individual consumers with firms, and of firms with other firms, as based on interwoven contingencies.

The import of this is that the marketing firm differs from many other commercial and social organizations not only by virtue of goal separation but also insofar as it has an output that is over and above that of the aggregated outputs of its members. This output, the marketing mix, is a set of discriminative stimuli (see Tarbox and Tarbox, 2017) and motivating operations that seeks to engender consumer behavior that is beneficial not only to the consumerate (in the form of utility maximization) but also to the firm (in the form of revenues and profits). Other organizations such as many cooperatives, partnerships, and social marketing campaigns have a goal which is identical to that of their members and an output that is the combined outputs of the membership. The kind of organization represented by the marketing firm is known as a *metacontingency* by reason of its have a superordinate behavioral output; its behavior, in putting out a marketing mix that is greater than the combined outputs of its members, may therefore be described as superordinate behavior. The behavioral output of the other organizations is known simply as *macrobehavior.* There is a crucial distinction. Behavior analysis usually takes the individual subject as its subject matter and insofar as the behavior of an individual is predictable from the three-term contingency, it may be understood as an “operant system.” The selection by the environment of the behavior of this individual has implications for the evolution of its behavior: behaviors that are reinforced are selected while those that are punished tend to die out. We are here treating an organization as an operant system. In the case of a metacontingency, its behavioral output is subject to selection by the environment in such a way that it evolves; this is not the case for macrobehavior. In the case of organizations whose output consists in macrobehavior, the concept of operant system applies only to its individual members; in the instance of a metacontingency, the organization at its center or hub is the operant system. The nature of a metacontingency can be better understood by considering it as a nexus of bilateral contingencies, and what Figure 3 therefore depicts is the corporate metacontingency as a nexus of bilateral contingencies.

The concept of the marketing firm rests on a distinction between the behavioral outputs of organizations that are metacontingencies and those of collectivities of persons who form the firm’s customer base (Biglan and Glenn, 2013; Foxall, 2015a,b) Thus the idea of a firm as a metacontingency derives from its behavioral output having emerged from, but existing over and above, the combined actions of its members, rendering the output of the metacontingency qualitatively different from the aggregated behaviors of its affiliates. Such metacontingent corporate behavior evolves in its own right as its consequences are selected or deselected by the environment, in this case by the firm’s customers and potential customers, who respond to the marketing mixes it presents. The behavioral output of the firm’s consumers is, in contrast, the aggregated consequences of their several actions. It may be possible to measure and statistically analyze this behavior but it does not thereby become an entity in its own right: it is simply combination of individual operant responses (Biglan and Glenn, 2013). This combined behavior does not therefore evolve: its increase or extinction is not sensitive to its environmental consequences since it produces no behavioral outputs that can be acted on by a selective environment. Biglan and Glenn (2013) describes these aggregated actions of a large collectivity as *macrobehavior*.

The marketing firm, however, generates a supra-personal output, the marketing mix – not just a product but a fusion of product and non-product elements by which the firm attempts to influence demand and which, acting as a single entity, may create a customer. The kind of firm we are considering is only competent to market successfully if it employs all four elements of the marketing mix in optimal fashion. Social marketing campaigns by contrast rely heavily on persuasive communication – in fact for many this is the sole element of the marketing mix employed. The deployment of a communications strategy is not marketing and clearly it does not entail marketing mix management.

#### Bilateral Contingency

**FIGURE 4:**
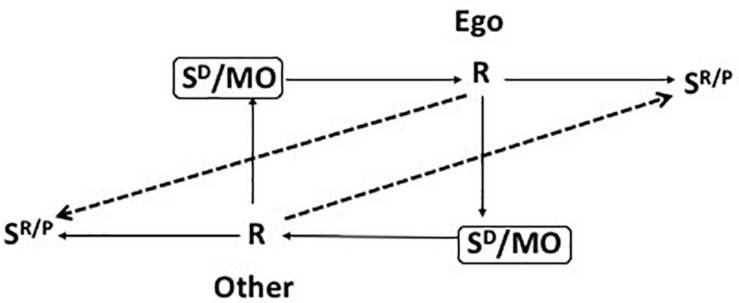
Bilateral contingency: the fundamental structure of a reciprocal relationship; Foxall (2020b).

The concept of bilateral contingency, introduced by Foxall (1999), captures the relationships between two individuals, each of whose behavior has implications for the structure of the other’s behavior setting. As Figure 4 indicates, bilateral contingency is a reciprocal interaction of the three-term contingencies that govern the behavior of the parties to a transaction or relationship. The behavior of one party supplies the reinforcing and punishing consequences of the other’s behavior; in addition, the behavior of one party provides discriminative stimuli and motivating operations for the behavior of the other. The meshing of these reciprocal contingencies determines whether the relationship between the parties is of short- or long-term duration; it therefore determines whether one or other party will engage in search for alternative arrangements.

Bilateral contingency was advanced first in the context of the exogenous corporate relationships that mark the interactions of the marketing firm with its consumerate and suppliers. There is growing recognition that the firm comprises a nexus of bilateral contingencies which potentially connect any and all of its members. Among the most significant, however, are those that link principal and agent. Such bilateral contingencies arise in the case of shareholders and directors, directors and managers, and managers and employees. Hence, the relationships between shareholders and directors, directors and managers, and managers and employees can also be interpreted as bilateral contingencies.

##### Extra-firm bilateral contingencies

The dominant theme of the firm’s external relationships is defined by the demands which its consumerate makes of it. The overall aim of the marketing firm is to create a consumerate. The “consumerate” encompasses the customer base of the marketing firm, be it composed of an aggregation of individual final consumers or a number of corporate customers. Drucker (1977, 2007) speaks of the objective of the firm as “to create a customer.” A customer is someone who purchases a product or service in sufficient quantity to enable the firm to fulfill its revenue and profit objectives; and a consumerate is that aggregation of the customer base that enables the firm, through repeat purchasing, to accomplish these goals. More formally, a customer is an individual or organization with which the firm interacts through marketing transactions (objective exchange, whole marketing mix deployment, pecuniary markets). Only firms (marketing firms) therefore have customers and by extension consumerates. Other organizations, even if they pursue commercial objectives, have publics but not customers or consumerates. Building on Drucker’s work, therefore, the objective of the firm – in the age of the imperatives – is the creation of a consumerate.

**FIGURE 5:**
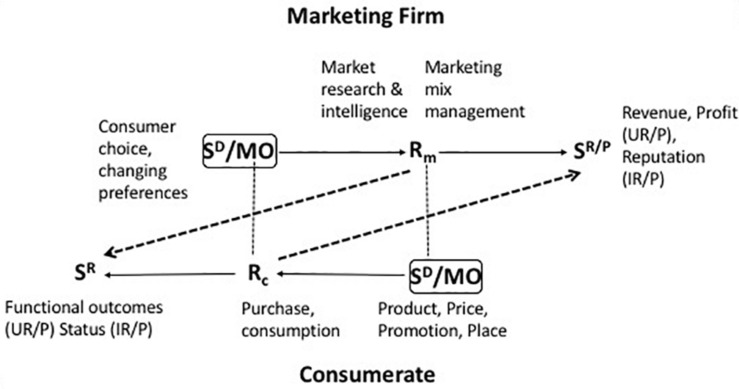
Extra-firm bilateral contingency; Foxall (2020a).

The firm’s behavior is summarized by the three marketing operations previously mentioned and described at greater length in Foxall (2018, 2020a), namely, (i) reading consumer behavior and preferences in the course of creating marketing intelligence and (ii) translating this into a strategy that consists ultimately in (iii) the provision and management of a viable portfolio of marketing mixes. Hence, at its simplest, the behavior of the consumerate acts as a discriminative stimulus for the firm while that of the firm is a discriminative stimulus for consumer behavior. More complexly, bilateral contingency analysis involves the overt relationships between the marketing firm and a customer, either a final consumer or a corporate purchaser (Figure 5). The bilateral contingencies linking firm, stakeholders, and consumerates include contractual relationships, e.g., between the firm and its customers, or the firm and its employees, and noncontractual connections among these parties, both commercial and social. These latter are “mutuality” relationships (Foxall, 1999).

The goal of marketing firms is to consider, create, and apply marketing mixes that profitably satisfy the firm’s consumerate. The components of the marketing mix (product, price, promotion and place utilities) appear in the marketplace initially as discriminative stimuli for the consumer behaviors of browsing, purchasing and consuming. Purchasing, the exchange of money for the ownership of the legal right to a product or service: such pecuniary exchange is a source of both utilitarian reinforcement (in the form of resources that can be paid out or reinvested) and informational reinforcement (in the form of feedback on corporate performance) for the marketing firm. The effectiveness of *R*_*m*_ (managerial behavior) in fulfilling the obligations of professional marketing management, specifically the creation of a customer who purchases the product at a price level sufficient to meet the goals of the firm, is governed by the generation of revenue/profit and reputation for the firm (depicted by the appropriate dotted diagonal line in Figure 5). This consumer behavior (*R*_*c*_) also provides discriminative stimuli for future marketing intelligence activities, marketing planning and the creation and execution of marketing mixes that respond to the constancies and/or dynamic qualities of the behavior of the consumerate (Foxall, 1999, 2014; Reed, 1999; Vella and Foxall, 2011, 2013). When looking at this particular level of interaction between the firm and its customer base, managerial behavior can be viewed as optimizing a utility function, comprising a combination of both utilitarian reinforcement and informational reinforcement. The firm is entrenched within a nexus of bilateral contingencies and the management of multilateral contingencies is at the center of its administrative task. That being said, the firm is not necessarily *coterminous* with such a nexus but it provides the nucleus of the network of interrelationships among its stakeholders. Similar bilateral contingencies mark the relationships between the marketing firm and its suppliers, in the analysis of which the supplier assumes the role of the marketing firm.

##### Intra-firm bilateral contingencies

The firm has been characterized as a nexus of contracts. In the modern corporation owners appoint the firms officers, managers who carry contractual responsibility to operate the organization in a manner that maximizes returns to these shareholders. Managers appoint (other) employees who are contractually responsible for conducting those business operations that fall within the domain of their assumed competence. These are all principal—agent relationships. In addition, managers interact with other managers and employees with employees in order to fulfill their responsibilities. These relationships all fall within the compass of *intra-firm bilateral contingencies*, a designation that is capable of embracing both the contractual relationships involved and the additional, “mutuality relationships” that lubricate these contractual interactions but do not fall within the scope of the contracts the parties have signed. This topic is important because of the argument (e.g., Singer, 2019) that while markets operate in a manner that is consonant with the values of liberal democracy, *within* the firm this is far from the case: managers exercise authority and workers are exploited; in other words, while firms operate within free markets their internal operations do not abide by market considerations but reflect managerial authority. If, however, the firm constitutes, essentially, a private market, as the Chicago School argues, we should expect to find that authority relations are less to the fore, if not entirely absent (Friedman, 1962). This debate is not an integral subject for the present paper, though the analysis of intra-firm bilateral contingencies is of relevance to it. For the present discussion, two assumptions are made. First, rather than behavior within the firm being determined by authority relationships, it is indeed, the result of individuals’ contracting together within a market framework (see, e.g., Alchian and Demsetz, 1972; Jensen and Meckling, 1976; Easterbrook and Fischel, 1991; Hart, 1995; Singer, 2019). However, there is in practice a degree of authority involved in the firm’s relations between principal and agent. The markets in which individuals operate as employees are not so smooth running as those between a consumer and her baker. An employee or manager can always leave their current position as per contract but an equivalent job is not necessarily to be had elsewhere without incurring considerable transaction costs (Larsen et al., 2020). But the principal-agent relationship is not an absolutely authoritarian one either. Of course, Friedman makes a good point: treating the firm as a private market makes it predictable, his 1953 criterion, even though the smooth jobs market envisaged requires many firms and competition among them (Friedman, 1953). More extreme perhaps is the assertion of the “property rights paradigm” (Alchian and Demsetz, 1972) to the effect that managerial power does not exist, that it is simply a contractual relationship, and that the institution that can direct the corporation is the market. This thinking does, however, have some resonance with the imperatives of customer-oriented management and the theory of the marketing firm.

We can designate the various linkages between firms and between members of firms in terms of their (i) direction (horizontal or vertical), (ii) contractual or non-contractual nature, and (iii) being marketing- or market-transactions, professional relationships, and mutuality relationships. Table 1 summarizes the possibilities for the marketing firm.

**TABLE 1:** Types of extra- and intra-firm bilateral contingency in the marketing firm.

	**Participants**	**Bilateral contingency**	**Type of relationship**
A	Marketing firm – consumerate	Horizontal, contractual, extra-firm.	Marketing transaction.
B	Supplier – marketing firm	Horizontal, contractual, extra-firm.	Marketing transaction.
C1	Shareholders – directors	Vertical, principal-agent, contractual	Market transaction.
C2	Directors – managers	Vertical, principal-agent, contractual	Market transaction.
C3	Manager – employee	Vertical, principal-agent, contractual	Market transaction.
C4	Manager – manager	Horizontal, non-contractual	Professional (mutuality) relationship.
C5	Employee – employee	Horizontal, non-contractual	Professional (mutuality) relationship.

**FIGURE 6:**
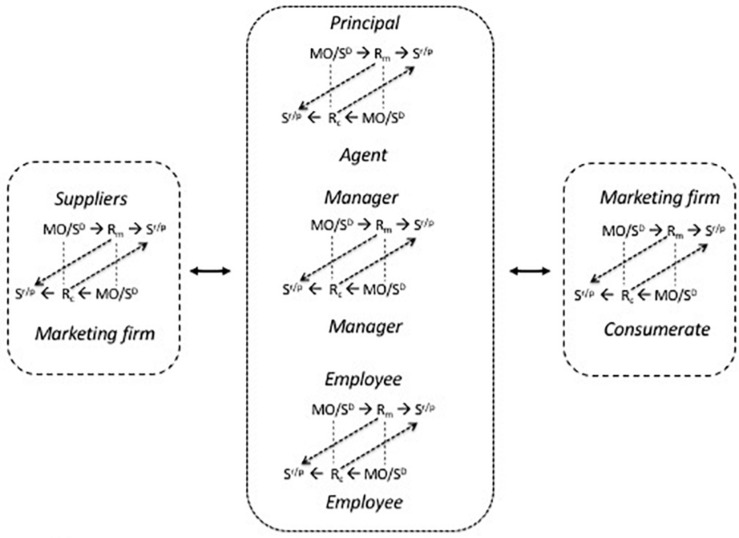
The marketing firm as the hub of a nexus of intra- and extra-firm bilateral contingencies; Foxall (in preparation).

While extra-firm bilateral contingencies involve marketing transactions (Figure 4) whether they are (A) from the marketing firm to a consumerate or (B) from a supplier to the marketing firm, and in both instances are horizontal, contractual relationships, intra-firm bilateral contingencies (C) are of various kinds. Those between shareholders and directors (C1) are vertical, principal-agent, and contractual; those between directors and managers are, similarly, vertical, principal-agent, and contractual (C2), as are those between managers and employees (C3). Those between manager and manager (C4) and employee and employee (C5) are horizontal and of themselves non-contractual. However, they may be enforced by general contractual obligations, especially in the case of manager-manager relationships based on superiority and hence sanctioned authority. A and B are marketing transactions: they are marked by the employment of a marketing mix that is comprehensively employed, pecuniary consideration, transfer of property rights, and competitive market-based pricing that entails contracting. C1, C2, C3 are market transactions: they are based on contractual relationships which in a competitive economic system permits the parties to sever their connections and seek employment or agents elsewhere. C4 and C5 are professional relationships, based for the most part on mutuality transactions which entail reciprocal consideration, interaction, co-working, and co-operation. However, they remain ultimately subject to contractual understandings. Disagreements at this point may not involve resort to the extra-firm employment market, however: there is no automatic need for an aggrieved person to resign. Resolution may be by managerial fiat and/or interpersonal accommodation. If C4 and C5 relationships operate smoothly it is likely to be because mutuality transactions work well. These are still bilateral contingencies, depending for their efficient operation on the appropriate rewards being available for work and on a system of discriminative stimuli and motivating operations that efficiently signal the appropriate behaviors in specific circumstances. These types of bilateral contingency are depicted in Figure 6 which also illustrates how the metacontingency that is the marketing firm is a nexus of bilateral contingencies.

Shareholders, directors, managers, and other employees are bound together by a range of mutuality relationships but, essentially, by more formal bonds that involve *inter alia* contracts of employment, legal requirements, and job descriptions. They determine and regulate literal exchanges, e.g., of remuneration and work done or investments made, and they occur in competitive markets (often labor markets), at market-determined prices. These are not, however, marketing transactions, since there is no marketing mix. They may, therefore, be understood as *market transactions.*

**Box 4. Meaning of “market transaction”**

A *market transaction* is a (i) contractual relationship which is legally governed, taking pace in a (ii) competitive market, and involving (iii) market prices/incomes. It does not involve the use of the marketing mix, however, and so is distinct from a marketing transaction (Foxall, in preparation).

Although, as Coase (1937) famously pointed out, incorporating as a firm overcomes some of the costs of transacting in the marketplace (searching for information, for suppliers, negotiating and policing contracts), there are administrative costs that arise when the owners or senior management, including the directors, of a firm are called upon to exercise control of the activities of the firm’s employees. The costs associated with this process include those of incentivization, information, and communication (Posner, 2014, p. 534): the employee in this situation is not paid in direct proportion to what he produces and thus has no incentive to reduce the costs incurred by his labor – he may moreover actively freeload on his co-workers; the employees are unaware of the prices paid for the resources they use, knowledge of which would tend to dictate the most profitable usages of such resources; the issuing of orders by top management and the monitoring of subsequent production requires an effective two-way means of communication. The costs involved in these activities in which the principal (shareholder, director, manager) seeks to maximize the efficiency of the agents (managers, employees) through whom the wishes of the principal are effected are known as agency costs; they are mainly incurred in the effort to maintain agents’ loyalty (Dnes, 2018; Zamir and Teichman, 2018).

The marketing firm stands at the hub of a metacontingency which embraces also the firm’s suppliers and its consumerate (Figure 6). Only the relationships included in the central entity marked by the heavier dotted lines comprise the firm, especially as it is legally defined. Organizationally, however, the boundaries of the firm are porous (Minkes and Foxall, 1982). The intra-firm bilateral contingencies brought into play by consideration of agency costs depicted as C1, C2, and C3 in Table 1 and Figure 6 are of this kind. They are vertical relationships that are at some level contractual, though the principal may not always be the direct party to the agent’s contractual obligation to the firm.

## Comparison With Consumer Organizations: Objectives, Functions, and Organization

### The Nature of the Charitable Organization

A charity is an organization that has the purpose of bringing about public benefit. A firm, by contrast, exists in order to benefit particular groups of people: its owners, directors, managers, employees and, in order to benefit these stakeholder groups, its customers. Even an organizations that exists to secure a more general benefit than this, such as a co-operative, is not a charity because it exists for the benefit of its members, a circumscribed goal that excludes much of the public. The sphere of operations of charities is specified in an English legal case of 1891, *Income Tax Special Purposes Commission v. Pensel* in which a charity is legally a trust for the relief of poverty, or the advancement of education, or for the advancement of religion, or finally for other purposes beneficial to the community (Gillard and Semple, 2018).

In the United Kingdom, for instance, a charity may have a variety of legal structures, existing as either a charitable incorporated organization, a charitable company that is limited by guarantee, an unincorporated association, or as a trust (Gov.UK, 2014). Internationally, charities come in many forms and seek to achieve diverse goals within the general rubric of being for the public good (Anheier, 2014). This account is, therefore, general in character. The questions it asks are not what does this or that specific charitable organization aim to do? but what is the goal of the charity as a whole in relation to the goals of its members? And in this regard how does it differ from the marketing firm? Not what does this or that specific charity do? what are its functions? but are charities capable of marketing-oriented management in the sense that this describes the firm? Not how is this or that charity organized? but how do the intra-organizational relationships among the various roles compare with those that typically describe the personal interactions within the firm? Finally, not what does the charitable organization achieve? but does the charity have a kind of output over and above the combined achievements of its various participants?

In economic terms, what serves to distinguish non-profits like charities from other commercial organizations is their financial structure: they are not allowed to distribute their profits or surpluses and they obtain most of their income not from selling products or services, nor through taxation, but through the voluntary giving of those who belong to and support them (Anheier, 2014, p. 70; see also Veludo-de-Oliveira et al., 2015).

Charities are clearly, therefore, not firms on this criterion. Moreover, a *marketing* firm has an objective, constrained sales-revenue maximization, which differs from the objectives of each of its stakeholders, whose motivation is presumed to be consumption. Hence, the marketing firm is a firm in Spulber’s sense. It shareholders, directors, managers, and employees may well, as he points out, *prefer* that the organization pursue an optimizing financial objective (profit maximization, in his theory), but their particular goals are summed up in the word consumption. The other defining characteristics of the marketing firm are inherent in its definition as that organization which responds to the imperatives of customer-oriented management. *Nonprofits*, including social marketing campaigns and charities, differ in several respects from marketing firms.

### Implications of Goal-Separation

Preceding sections established that marketing firms uniquely engage in marketing transactions, responding to market-determined prices, competing with other marketing firms, and employing the complete marketing mix as a unified means of achieving consumer sales. In addition, marketing firms have a clear-cut entrepreneurial or strategic process, responding to the imperatives of customer-oriented management, and participate in metacontingencies. In this section we are concerned to establish whether charities (as typical of non-profits) can be said to be either firms in Spulber’s sense or marketing firms in the sense established in this paper. The goals of non-profits, such as *charitable organizations* are coterminous with those of their stakeholders. It is unlikely that anyone would volunteer to work for such an organization, freely assigning their time and energy to it, without implicitly and explicitly sharing its humanitarian goal. Paid employees, in the even there are any, are also likely to embrace this objective since it is likely they will receive lower remuneration than they might elsewhere. Those who donate physical products to charities clearly also share their goals.

What distinguishes nonprofit firms in general is that they are not legally entitled to allocate any excess of revenues over costs. Hence, as Posner (2014, pp. 537–8) points out, if the nonprofit firm can be said to be maximizing anything, it is clearly something other than profit. The theory of the marketing firm comprehends this eventuality by assuming that all firms maximize a combination of utilitarian *and* informational reinforcement. It is clear, however, that nonprofits, including charities, can face important financial challenges. Posner notes that those nonprofit firms that engage in pecuniary exchanges, for instance, may not be able to price lower than for-profits since they still have to acquire capital in a competitive marketplace (and, we may add, labor too). *Charities*, however, fall into a special category because their capital and much of the labor they employ is donated to them: they can thus distribute their output at a low price or even give it freely to the recipients. The utility functions of charities’ altruistic donors contain non-monetary benefits that compensate them for the financial returns they could obtain by investing elsewhere. This behavioral preference on the part of those who donate labor and capital to the charity is central to the form such organizations assumed. Moreover, Posner views the donors to the nonprofit firm as akin to the shareholders of the for-profit firm. He also suggests that if the donors are not in control of the nonprofit firm’s board of directors and the board is not in need of new donations, problems of agency costs may arise, just as in for-profit firms (Posner, 2014, p. 538). Being an employee of such can often be very similar to working for a for-profit organization – a job is a job, a career a career. Those who donate physical items and their labor to nonprofit firms may not, of course, be pure altruists (i.e., those who give of their substance, seek nothing in return and indeed receive nothing in return). Donors may seek important informational reinforcement-based benefits such as the enhancement of their reputation as altruistic givers, the exhibition of the donor’s wealth, gaining publicity, and perhaps thereby promotes profitable commercial interests. Those who work for charities may gain self-esteem or pride, a raison d’être, social status among friends and the general public, positive self-references for resumés, and so on. In the case of religious charities they may even seek salvation and a happy afterlife and be rewarded here and now by assurance thereof.

Indeed, charities occupy an intriguing position in the matrix of consumer organizations considered here. As Corry (2014, p.5) points out, “The charity sector will never be like other sectors. Indeed its special role means we don’t want it to be. It is driven by a sense of mission and passion – few are involved to maximize profits, improve share prices or earn as much as they can.” A vital difference between charitable organizations on the one hand and the firm and other consumer organizations on the other is that charities do not seek to attain any specific response from the publics they serve. There is no sense of exchange; rather, there is a uni-directional acquisition (from donors) and provision (to recipients) of a product or service. Even religious charities are bound by this ethos, differing from religious organizations *per se* which seek to inspire a response in terms of conversion or membership from those to whom they appeal (one cannot rule out this motive from religious charities, of course, whose broader objectives, gained from the religious organizations that sponsor them, may include proselytization). By contrast, charitable organizations engage in a form of symbolic non-literal exchange with those from whom they seek to elicit donations but the relationship is one of mutuality rather than transactive marketing.

### Implications of Market-Based Pricing and Competition

Charities are not firms: since they are non-profit entities, they clearly are not profit-maximizers, nor yet sales-revenue maximizers. Nor can they be said to practice goal-separation since their corporate objective, the provision of free (at the point of provision) aid to citizens, is identical to that of each of their stakeholder groups. nor yet do they share important characteristics with marketing firms. They are, in particular, unable to practice full customer-oriented management since their publics are identified and defined on the basis of demand, which may come from a variety of sections of the population which cannot necessarily be delineated prior to their appeal for assistance. In particular, their provision of charitable service is not governed by a price mechanism since it is unreservedly given.

A marketing mix includes a product or service, communications strategies, distribution management, and market-determined pricing; the marketing firm seeks to manage each of its marketing mixes as a unified whole that generates consumers and sales, understood as long-term buyers of the marketing firm’s productive outputs and repeated purchases. The insistence of competitive market-based prices is consistent with members of the firm’s consumerate having the discretion to allocate their discretionary income efficiently among the products and services available in the market-place. Market-based prices are also capable of indicating to the marketing firm the productive enterprises in which it should engage and those of which it should divest itself.

Among firms and consumer organizations, only the marketing firm unequivocally faces market-determined prices. Marketing cooperatives have a large say in determining prices by virtue of the countervailing power they can exercise by reducing competition among suppliers and acting quasi-monopsonistically. Hence, it seems that marketing cooperatives cannot be considered organizations that are governed by market prices. Some professional partnerships may operate in markets that determine prices but others are regulated in what they can charge by professional bodies. Hence, professional partnerships may face market pricing under circumstances to be delineated by empirical investigation. In the case of public corporations, exposure to market price mechanisms depends on the nature of any involvement of government or quango. Similarly, in such organizations, entrepreneurial and strategic operations may be limited.

Moreover, only the marketing firm unequivocally faces market competition, though marketing cooperatives may well face competition from marketing firms operating in the same fields and, possibly, from other cooperatives, though such businesses are usually geographically confined. Professional partnerships may compete with similar organizations, though they may be regulated so that they cannot overtly compete (e.g., restrictions on advertising and promotion).

### Implications of the Entrepreneurial (Strategic) Process

Managing the strategic process of the firm is the central task of the officers of the marketing firm and serves to differentiate their endeavors from those of the managers of consumer organizations. This management task is that of determining the firm’s strategic scope, defined by the range of marketing mixes the firm is able to support and the spectrum of consumerates through whose service it can achieve its objectives of survival and profit-maximization. Achieving a strategic scope that attains these objectives involves the performance of three marketing operations and each of these entails specific informational inputs and decision outputs. These operations are the *Creation of Marketing Intelligence* which consists in market search and appropriate managerial response to reveal the potential (*feasible*) strategic scope of the firm given its capabilities and resources; the *Formulation of Marketing Strategy*, through which is determined the planned strategic scope of the firm, namely the markets it proposes to serve, the nature of the marketing mixes with which it will do so, and the necessary diversification and innovation this requires. This procedure decides the *potential* strategic-scope of the firm and is based on detailed planning of the product-market scope of the firm and its resourcing; and *Marketing Mix Management*, in which the resultant portfolio of marketing mixes is planned, constructed and implemented through its life cycles. The outcome of this managerial activity in which the firm’s offerings are brought to the consumer who decides her action by buying or rejecting them demonstrates the *revealed* or *effective* strategic scope of the firm.

Consumer organizations have no need of these marketing operations in order to achieve their consumption goals. Unlike the marketing firm, there is no need to respond to marketing incompleteness. Their markets are given by the purpose which originally brought them into being, namely the consumption behavior of the publics they serve. They have no need of strategic scope analyses and administration. A charitable organization has no need of a profit goal of any shape or form since it is either supplied by voluntary donations or publicly funded; it is unable to deploy a comprehensive marketing mix because it is not subject to a price mechanism and its “product” or “service” is not exchanged in a literal or objective manner that transfers property rights between the parties to a transaction. There is no direct competition involved in its actions (indeed, the only “competition” that can be envisioned within its sphere of operations is that for the consumer’s time from all other sources of activity in which she might engage – hardly a precise definition). There is no reason why such an organization needs to be entrepreneurial since its sphere of operations is given and it has no incentive to innovate or alter either its offering or its consumerate: indeed, there may be, as a result of its remit, strict limitations on its changing either. There is no need to explore new “markets” within a charitable organization. Such an organization is neither a firm nor a marketing firm.

**FIGURE 7:**
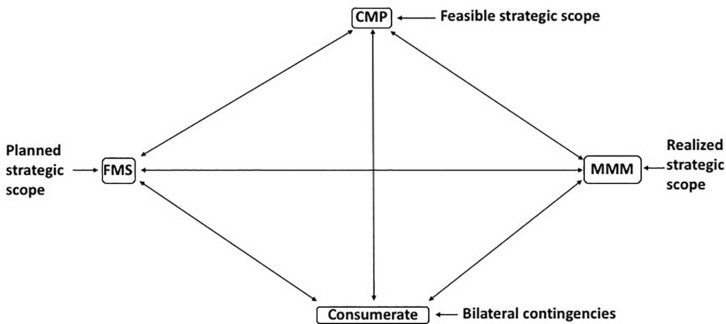
The strategic (entrepreneurial) process encompasses the consumerate. CMI, creation of marketing intelligence; FMS, formulation of marketing strategy; MMM, marketing mix management; Foxall (in preparation).

A major point of differentiation between marketing firms and consumer organizations of all kinds lies in the marketing firm’s incorporation of the consumerate in the entrepreneurial process (Figure 7). Many business-to-business firms co-invent and co-innovate with customers for instance; and many business-to-consumer firms are also keen to use consumer insights and to market test and test market along with customers. The consumerate is an integral part of the strategic process. On the face of it only marketing firms have incentive and opportunity to include their consumerates in the entrepreneurial process. However, although its scope is limited in this regard, the marketing co-operative has restricted ability to vary its product-market scope, pursue strategic marketing, or marketing mix management. The question is how far this reduces the gap between the marketing firm and the co-operative in this context. Moreover, partnerships may have some scope for this, albeit professional regulation may restrict their scope (for instance, some professional partnerships are restricted by their governing institutes from offering services jointly with other professions). The difference in managerial perception, perspective, and operations is, however, immense.

### Implications of Customer-Oriented Management

The marketing firm is unique in seeking to discover marketing incompleteness and to respond to it by the creation and management of a portfolio of marketing mixes that respond to the realities of consumer discretion and consumer sophistication. This is the mechanism by which it maximizes sales revenue and earns the profits necessary to satisfy its stakeholders and to reinvest in the business. As customer requirements change, so the business changes by adapting its mission and sphere of operations. The consumerate is the determinant of what business the firm is in and, equally important, the firm is free to respond by changing its strategic mission. There is no corresponding impetus for the charity which is locked in to a particular mission and function: to depart from this it would cease to exist. Firms may diversify; charities, not. The firm is an instigator of markets and marketing opportunities; the charity, a response to a specific need. The firm’s success in carrying out its mission is apparent from its levels of sales and profitability, dynamic market signals that determine the direction and scope of the firm; these attainments also put a limit on the activities of the firm by enhancing or circumscribing its sphere of operations for instance. The charity is limited to subjective feedback on its operations; it has no market signals to propel it into new directions.

### Implications of Organization

**FIGURE 8:**
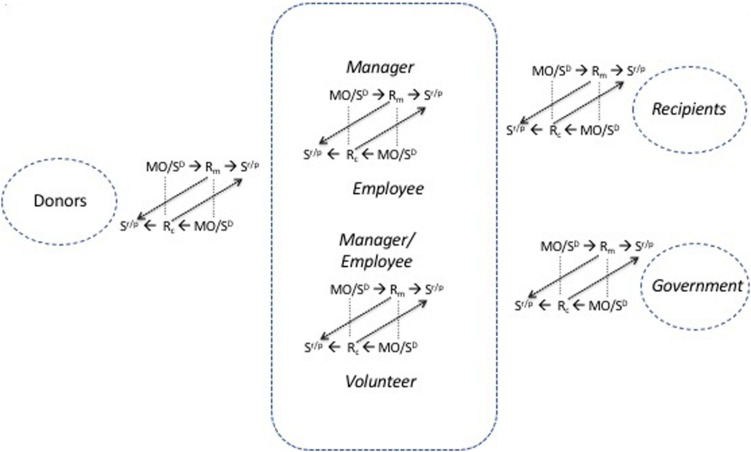
Bilateral contingency: the charity; Foxall (in preparation).

The relevance of bilateral contingency to the marketing firm was discussed above. Here we compare the marketing firm in terms of bilateral contingency with consumer organizations. Non-profits are characterized by the relationships shown in Table 2 (in the case of charities) and Figure 8. Although charitable organizations differ markedly depending on social, cultural, and legal contexts (e.g., Chevalier-Watts, 2017), some generalizations can be suggested. Crucially, what is missing from both the table and the figure is mention of C1, which in the case of the marketing firm represents a relationship in which shareholders’ controlling the directors, even to the extent of having statutory influence. In the instance of the charitable organization, the donors may as Posner suggests *resemble* shareholders and it is true that in some circumstances they may be in a position to exert influence and control. However, many charitable trusts came into existence already in possession of considerable endowments from donors which they retain. There is a strong likelihood that the donors may be unable to exert any pressure on the board of trustees or directors, and therefore on the managers, if no further donations are required to ensure the survival and prosperity of the organization.

**TABLE 2:** Types of bilateral contingency: the charity.

**Relationship**	**Participants**	**Bilateral contingency**	**Type of relationship**
A	Charity – donors	Horizontal, non-contractual, extra-organizational	Gift/near gift (mutuality) relationship
B	Charity – recipients	Horizontal, non-contractual, extra-organizational	Gift/near gift (mutuality) relationship
C2	Board – managers	Vertical, principal-agent, contractual	Market transaction
C3	Manager – employee	Vertical, principal-agent, contractual	Market transaction
C4	Manager – manager	Horizontal, non-contractual	Professional
C5	Employee – employee	Horizontal, non-contractual	Professional

Non-profits often and social marketing campaigns and charities generally meet their publics through *gift or quasi-gift relationships*. These are a not marketing transactions since the consideration, though often involving physical products or legally constituted services, are not paid for, certainly not at market rates. Objective exchange is unlikely in these circumstances; nor is a competitive pecuniary market likely, though there may be some form of token pricing. Usually, however, there is some form of subsidization, possibly by government, possibly private. There is no market price involved and on those grounds alone there can be no full marketing mix. The product or service involved is provided freely or almost freely and it is on these grounds that we refer to this as a gift or near-gift relationship. The supplier might be a commercial firm, government, or a private donor. This situation does not involve marketing transactions of any kind.

## Discussion and Conclusion

The marketing firm differs from consumer organizations in four respects. First, as we have seen, in respect of its goal separation, its having a goal (profit-maximization) that is distinct from those of its stakeholders. By contrast, consumer organizations pursue consumption goals on behalf of their members; this is true of cooperatives, partnerships, welfare organizations, social marketing campaigns, and public corporations; second, by responding to the imperatives of customer-oriented management, consumer choice and consumer sophistication. It is, specifically, its responding to the imperatives that makes the firm a *marketing* firm. Consumer organizations have their mission set for them. This is especially the case for public corporations and social marketing campaigns but also for cooperatives, and some welfare organizations. They are not free to shift their assets into serving wholly different product-markets – admittedly not many firms do this, but they are always legally free to do so. Third, marketing firms are unique in engaging in marketing transactions based on literal or objective exchanges of property rights, deployment of the whole marketing mix, operating in pecuniary markets, and facing competitive market prices. Consumer organizations, by contrast, lack competitive market prices *or* do not employ entire marketing mixes; moreover, the exchanges in which they participate are often not objective, being at the most symbolic. They are linked to their publics via bilateral contingencies but not by markets. Finally, marketing firms practice entrepreneurship through dynamic alertness to the opportunity for profit through better serving existing consumer needs or by satisfying needs new to the firm (e.g., Kirzner, 1973; Holcombe, 2007).

The result is a portfolio of marketing mixes that achieves the objectives of the firm, its consumerate, and its stakeholders. This array of marketing mixes generated and managed by marketing firms constitutes an output that is over and above the combined outputs of their members; upon the reception of these marketing mixes by the market depends the firm’s ability to achieve its objective. The marketing firm therefore participates in a *metacontingency*. Consumer organizations do none of these (Foxall, 2020a). Rather, they promote the consumption potential of their stakeholders, namely the income, pecuniary and/or psychic these individuals receive which increases their opportunities to consume. Charities specifically differ in that they may have the advantage of offering lower or zero prices to their publics. The theory of the marketing firm, based on a synthesis of behavioral psychology, microeconomics, and marketing science, allows these differences between marketing firms and consumer organizations to be understood within a common framework of conceptualization and analysis. This has important implications for how researchers view the nature of managerial and non-managerial work in these various kinds of business organization. Whilst we can treat pecuniary reinforcement as both utilitarian *and* informational reinforcement (as money or income serves both functions in Hertzberg’s two-factor theory: see, e.g., Herzberg et al., 1959; Herzberg, 1964, 1966), we must recognize that in some consumer organizations, such as charities this may be small or nonexistent. But as we have seen charity workers receive income in the form of informational reinforcement, the pride and so on they gain from participating. We need the apparatus of bilateral contingency to do justice to this, especially the concept of mutuality relationships as well as marketing transactions. The marketing firm moreover is a *hub* of bilateral contingencies. Unlike most consumer organizations it *participates in a metacontingency.* It has an output which is over and above the combined outputs of its members. Consumer organizations do not: their output takes the form of macrobehavior. In addition, the marketing firm, by definition, responds to the imperatives of customer-oriented management by generating its profits by means of the fulfillment of considerations arising from consumer choice and consumer sophistication. As a result the nature of managerial work in the marketing firm differs profoundly from that of consumer organizations: the necessity of strategic entrepreneurship on the part of the marketing firm is the key. Finally, we can extend the theory of the marketing firm by taking consumer organizations into consideration with respect to (i) intra-organizational bilateral contingencies (their incidence and strength) (ii) by comparing the marketing firm’s participation in a metacontingency with the capacity of consumer organizations to generate only macrobehavior (iii) by introduction of the concept of *multilateral contingency* which is capable of uniting analyses of the marketing firm and consumer organizations. Further research might concentrate on some apparent differences between marketing firms and consumer organizations that call for empirical investigation. In the marketing firm, for instance, goal separation imposes a hierarchy extending from shareholders (owners) to directors and then managers to employees. What are the implications for the range of bilateral contingencies that this entails for the management of the organization? What is the link between the marketing transactions that make up some of these contingences and the mutuality relationships that support them? Is it the case that consumer organizations generally have a much simpler and shorter chain of command than marketing firms? How does principal–agent management differ in organizations that include shareholders, directors, managers, and employees from those that involve trustees and directors, managers, and volunteers, and how do these various relationships differ in terms of day-to-day management and the monitoring and control of supervised organizational members? A further possibility for empirical investigation arises from a specific observation about charitable organizations made by Posner and adopted in this paper. It concerns the situation in which donors (treated as quasi-shareholders) do not control the board of trustees, and the board has no need to introduce new capital into the organization (perhaps by virtue of the large endowments that were provided when the charity was established), then agency costs are likely to be incurred through a lack of incentive to garner new donors or otherwise satisfy the charity’s publics. The assertion is that directors and managers may therefore slack off from the pursuit of high levels of efficiency as long as the minimal goals of the organization are met; far from being maximizers, they have become low-level satisficers. Can we adduce case study evidence for this and also gain some general idea of how prevalent these occurrences are among charities?

In conclusion: there is great potential for further research based on the discussion in this paper. Further analysis of charities by a deeper investigation of their objectives, practices, outcomes, and organizational structures would be beneficial in increasing understanding of their relationship with their markets. It is also recommended that the analysis of consumer organizations is extended by considering, in addition to charities, co-operatives, partnerships, state-owned enterprises. These additional research streams will add to a better understanding to consumer organizations as a whole.

## Author Contributions

GF inaugurated the project, did primary research into the theory of the marketing firm and the nature of charitable organizations, and wrote the earliest drafts. All authors contributed to the writing of the final draft. All authors contributed to the article and approved the submitted version.

## Conflict of Interest

The authors declare that the research was conducted in the absence of any commercial or financial relationships that could be construed as a potential conflict of interest.
